# Temporal Response of Endogenous Neural Progenitor Cells Following Injury to the Adult Rat Spinal Cord

**DOI:** 10.3389/fncel.2016.00058

**Published:** 2016-03-09

**Authors:** Yilin Mao, Kathryn Mathews, Catherine A. Gorrie

**Affiliations:** Neural Injury Research Unit, School of Life Sciences, Faculty of Science, University of Technology SydneySydney, NSW, Australia

**Keywords:** spinal cord injury, neural progenitor cells, reactive astrocytes, astrogliosis, temporal response, critical time period, Nestin, GFAP

## Abstract

A pool of endogenous neural progenitor cells (NPCs) found in the ependymal layer and the sub-ependymal area of the spinal cord are reported to upregulate Nestin in response to traumatic spinal cord injury (SCI). These cells could potentially be manipulated within a critical time period offering an innovative approach to the repair of SCI. However, little is known about the temporal response of endogenous NPCs following SCI. This study used a mild contusion injury in rat spinal cord and immunohistochemistry to determine the temporal response of ependymal NPCs following injury and their correlation to astrocyte activation at the lesion edge. The results from the study demonstrated that Nestin staining intensity at the central canal peaked at 24 h post-injury and then gradually declined over time. Reactive astrocytes double labeled by Nestin and glial fibrillary acidic protein (GFAP) were found at the lesion edge and commenced to form the glial scar from 1 week after injury. We conclude that the critical time period for manipulating endogenous NPCs following a spinal cod injury in rats is between 24 h when Nestin expression in ependymal cells is increased and 1 week when astrocytes are activated in large numbers.

## Introduction

Traumatic spinal cord injury (SCI) arises from a mechanical insult that damages the spinal cord. People living with SCI have symptoms that can include a range of sensory and motor deficits, chronic pain, bladder/bowel dysfunction as well as autonomic and sexual dysfunction (Norenberg et al., 2004). These reductions in function can affect quality of life for those suffering from the injury, and can also have an impact on society, with the families and the community, bearing many of the emotional and financial costs of SCI. At present, there are no fully restorative treatments for SCI, although various molecular, cellular and rehabilitative therapies have been approaching clinical trials and have shown a limited degree of functional recovery. Unfortunately, the existing clinical approaches for SCI are still limited in the short term (Thuret et al., 2006), with long-term functional outcomes largely unchanged despite the distinct improvement in the acute mortality rate (DeVivo, 2007). Consequently, it is critical to develop novel approaches for long-term functional recovery from SCI. Manipulation of neural progenitor cells (NPCs) is a promising avenue of approach.

NPCs are multipotent and able to differentiate into neurons, oligodendrocytes and astrocytes *in vitro* (Dromard et al., 2008). They are capable of self-renewal, proliferation and differentiation, although these responses are more limited and weaker compared with pluripotent stem cells (Mothe et al., 2011). NPCs are found throughout the mammalian central nervous system (CNS), including the spinal cord (Mothe and Tator, 2005; Mothe et al., 2011). These cells show promising results in a range of animal models investigating functional recovery after SCI (Mothe and Tator, 2005; Cizkova et al., 2009; Mothe et al., 2011; Faulkner et al., 2013). There are two principal ways in which NPCs may be used; exogenous NPCs can be transplanted into an injury site, or endogenous NPCs could be manipulated *in situ* for spinal cord repair. Both approaches, however, focus on utilizing the neuroregenerative capacity of NPCs to replace damaged or lost cells in the injured spinal cord. The manipulation of endogenous NPCs as a minimally invasive therapy may be preferable for treating SCI, because it can circumvent key issues associated with stem cell transplant, such as immune responses and cell rejection, tumorigenesis and ethical issues regarding the sourcing and transplanting of stem cells (Barreiro-Iglesias, 2010).

In the normal uninjured spinal cord, endogenous NPCs are quiescent in the ependymal and sub-ependymal areas of the central canal (Shibuya et al., 2002; Hamilton et al., 2009). However following injury endogenous NPCs in the spinal cord proliferate, migrating to the lesion site for differentiation (Hofstetter et al., 2005; Horky et al., 2006; Meletis et al., 2008; Cizkova et al., 2009; Barnabé-Heider et al., 2010). After migration, the majority of the activated NPCs differentiate into astrocytes, contributing to glial scar formation (Ke et al., 2006; Vessal et al., 2007; Barnabé-Heider et al., 2010). This glial scar, while protecting the injury site from further damage, also inhibits the repair of the injury by creating a physical and chemical barrier. Spinal cord NPCs are capable of differentiation into all three neural lineages, neurons, oligodendrocytes and astrocytes *in vitro* (Blasko et al., 2012), but there is currently no evidence of spontaneous neurogenesis from endogenous NPCs *in vivo* (Ke et al., 2006; Vessal et al., 2007). NPCs can differentiate into neurons *in vivo* given the right conditions as shown by a recent murine study that introduced the transcription factor Sox11 gene via a lentiviral vector (Guo et al., 2014). This treatment pushed NPCs towards a neuronal lineage, increased brain-derived neurotrophic factor expression and resulted in improved functional outcomes. Sox11 activation is associated with an up-regulation of Nestin protein in ependymal cells following SCI. Nestin, a type VI intermediate filament protein, predominately presents in CNS progenitor cells (Tzeng, 2002), and is also expressed by the ependymal cells in the central canal of developing spinal cord and in response to traumatic injury (Sakakibara et al., 2007; Cizkova et al., 2009). In our study, NPCs were recognized by the immunohistochemistry antibody, anti-Nestin, with their histological location around the central canal (Cizkova et al., 2009). We have shown that Nestin is increased in human spinal cords following injury to the CNS, (Cawsey et al., 2015), but it can be difficult to determine the temporal sequences using human autopsy tissue, with all its inherent variation. Although several studies have reported increased Nestin expression in rat models of SCI (Namiki and Tator, 1999; Shibuya et al., 2002; Mothe and Tator, 2005; Horky et al., 2006; Cizkova et al., 2009; Foret et al., 2010), there remains some uncertainty about the time of peak response. We propose that a therapeutic window exists while NPCs are expressing Nestin but before cells are committed to astrocyte differentiation.

## Materials and Methods

### Animals

Twenty one adult female Sprague-Dawley rats (250 ± 10 g) were used (Animal Resources Centre, Perth, Australia). Rats were maintained in standard cages with ad-lib water and food on a 12:12 h light-dark cycle. Rats underwent SCI, and were euthanased at different time points post-injury, 24 h, 1 week, 2 weeks and 6 weeks. Three rats were subjected to sham surgery (laminectomy only) and kept for 6 weeks following surgery. Six normal rats were sacrificed at 0 h and 6 weeks as controls (*n* = 3 for all groups). All animal procedures were in accordance with the guidelines of the National Health and Medical Research Council of Australia, and the experimental protocol was approved by The University of Technology Sydney Animal Care and Ethics Committee.

### Surgical Procedures

Anesthesia was induced using 4% Isofluorane with 1 L/min oxygen and then maintained at 2% Isofluorane with 1 L/min oxygen. Skin and muscle were incised and retracted through the dorsal midline and a T10 laminectomy was performed to expose spinal cord. A New York University Weight-Drop Impactor was used to induce a mild contusion SCI (6.25 mm drop, 10 g weight, 2.5 mm diameter). The surgical data, including compression (mm), compression rate (m/s) and weight-drop velocity (m/s), was recorded by the impactor software. The surgical wound was then closed in layers. Rats were given antibiotics (Cephalozin sodium, 33 mg/kg, s.c.), analgesics (Temgesic 0.03 mg/kg, s.c.) and supplementary fluid (Hartman’s replacement solution, 15 ml/kg, s.c.) post-operatively twice daily for 3 days. Bladders were expressed manually until the normal voiding response returned. Antibiotics were continued until the urine was clear. The skin suture was removed 7–10 days post-operation.

### Behavioral Assessments

Each animal was assessed for functional recovery on post-operative day 1 only using the BBB Locomotor Rating Scale to ensure consistency of the lesion (Basso et al., 1996). Assessment was carried out by two independent reviewers who were blinded to the groups.

### Histology

Animals were deeply anesthetized by an injection of Lethobarb (Pentobarbitone sodium, 1 ml/kg, i.p.), and then perfused intracardially with 0.9% saline with heparin, followed by 4% paraformaldehyde in 0.1 M phosphate (pH 7.4). The spinal cord was collected from cervical enlargement to lumbar enlargement for 24 h fixation in 4% paraformaldehyde, and then cryoprotected in 30% sucrose in 0.1 M phosphate buffer with 0.01% sodium azide. Transverse sections were produced at 15 μm from a 1 cm length of spinal cord centered on the injury epicenter. All the sections were mounted in series so that adjacent sections were on consecutive slides. One slide of each spinal cord was stained with Mayer’s Haematoxylin and Eosin (H&E) to assess tissue morphology and determine the injury site.

### Immunohistochemistry

One slide of each spinal cord was used for co-labeling of Nestin and glial fibrillary acidic protein (GFAP). Slides were taken through xylene and decreasingly graded ethanol to distilled water. They were then immersed in phosphate buffered saline with 0.2% Triton X-100 (PBST) at pH 7.4 for 10 min, followed by 5% normal goat serum in PBST for 30 min. Primary mouse anti-Nestin (Abcam, 1:1000) and rabbit anti-GFAP (Dako, 1:1000) antibodies were diluted with phosphate buffer with 5% normal goat serum (PBG), and applied on slides. Primary antibody was omitted on negative control slides. All slides were incubated overnight at 4°C in a moist environment. Three changes of PBST were used to wash the slides. The secondary Alexa Fluor fluorescent 568 anti-mouse (Invitrogen, 1:200) and 488 anti-rabbit (Invitrogen, 1:200) antibodies diluted with PBG were applied on slides. All slides were incubated for 2 h at room temperature in a moist environment. For nuclear staining, slides were washed by two changes of PBST and then incubated in 0.2% Hoechst (Invitrogen) for 10 min. All slides were coversliped in fluorescent mounting medium (Dako).

### Image Analysis

All sections were captured using Olympus DP70 camera with the Olympus DP Controller software (Version 3.1.1.267). Entire spinal cord sections were captured at low power (40×) and regions of interest (ROI) were also captured at high power (400×).

ROI (0.01 mm^2^) included:

- edge of the lesion;
- center of the lesion;
- ventral white matter and ventral gray matter;
- central canal.

Images were captured on sections containing the lesion epicenter and 2.25 mm rostral and caudal to the epicenter for each injured spinal cord. The equivalent sections were selected in sham and control groups for imaging.

Injury area was identified as hemeorrhage, axonal swellings, cell death and cavity formation on H&E images. The transverse area of spinal cord and the injured area were measured on every H&E section using ImageJ Software (National Institutes of Health, Version 2.1.4.7). The injury percentage was calculated as the injured area divided by the transverse area of spinal cord to determine the section containing epicenter of injury (maximum injury percentage).

Nestin and GFAP immunoreactivity was measured in each ROI using the mean gray scale value (GSV) as a measurement of staining intensity. For fluorescent microscopic images the black balance of images was adjusted to the level of negative control as reference by the build-in function of the software (400× magnification, U-MWIG3 filter, 1/10 s exposure time) to control for any variation in staining intensity between sections or staining runs.

### Statistics

Data analysis was performed using GraphPad PRISM Statistics Software (Version 6.0e). All data was expressed as mean ± standard deviation (*n* = 3 for all groups). Statistically significant differences were considered at *p* < 0.05. Pearson’s correlations were employed to evaluate the relationship between Nestin and GFAP immunoreactivity. The Pearson’s correlation coefficient (*r*) indicates a linear relationship (Welkowitz et al., 2012). Analysis of variance (ANOVA) was used to compare between the groups and Bonferroni *post hoc* tests were used to determine significant differences. Paired *t*-tests were used to compare caudal and rostral measurements within in the same animal.

## Results

### Contusion Injury

The mild contusion SCI was produced consistently in all injury animals according to surgical parameters and day 1 BBB scores. There were no significant differences in weight-drop velocity, compression and compression rate between the four injury groups (ANOVA, *p* > 0.05). The mean weight-drop velocity, compression, and compression rate were 0.348 ± 0.028 m/s, 1.707 ± 0.152 mm and 0.301 ± 0.007 m/s respectively. In terms of the hindlimb locomotor function at day 1 post-surgery, there were no significant differences in day 1 BBB score between the four injury groups, or between 6-week sham and normal control groups (ANOVA, *p* > 0.05). The mean BBB scores at day 1 post-surgery were 4.8 ± 2.5 for the injury groups and 20.9 ± 0.2 for the 6-week sham and normal control groups.

### Lesion Morphology

**Figure 1:**
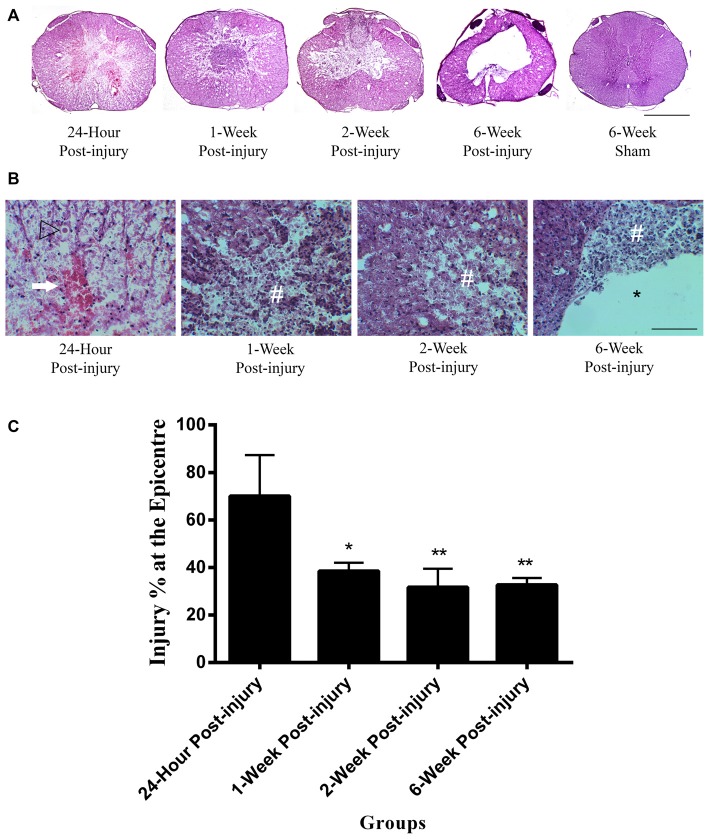
**Lesion epicenter in rat spinal cord transverse sections. (A)** Mayer’s H&E staining of lesion epicenter at different time points. The lesion induced by the mild contusion was observed at the dorsal site of the spinal cord in the injury groups. The central canal in the injury groups was destroyed at the epicenter of injury. Scale bar: 500 μm. **(B)** Mayer’s H&E staining of lesion area at high power at different time points. Hemorrhage (arrow) was extensive at 24 h post-injury and reduced by 1 week following spinal cord injury (SCI). Extensive axonal injury with axonal swellings and retraction bulbs (arrow heads) were visible in all injury groups. Necrotic tissue and cellular debris (#) were observed in the injury groups. Cell loss in the lesion increased over time until a cavity (*) formed by 6-week post-injury. Scale bar: 125 μm. **(C)** Percentage of the injured area at the epicenter of injury between injury groups. The 24 h injury group had a higher injury percentage at the epicenter of injury than other injury groups. Data are presented as mean ± SD. Statistically significant differences were shown compared to the 24 h injury group. *p < 0.05, **p < 0.01.

The mild contusion led to a lesion located in the dorsal surface of the spinal cord (Figure 1A). In injury groups, the central canal at the epicenter of the mild contusion was completely fragmented, and the individual ependymal cells could not be recognized. There was extensive axonal injury in the white matter with axonal swellings and retraction bulbs visible in all injury groups from 24 h post-injury. Axonal swellings and retraction bulbs are easily identifiable on H&E stained transverse sections of spinal cord (Figure 1B) and appear as small round eosinophilic structures located in the white matter. The lesion site involved necrotic tissue, cellular debris in 24 h, 1-week and 2-week post-injury groups. Hemorrhage was extensive at 24 h post-injury and reduced by 1-week following SCI. There were a large number of neutrophils, clearly identifiable by their multi-lobed nuclei, spreading across the lesion and surrounding area at 24 h post-injury, suggesting acute inflammation, and they remained in small numbers at 1 week post-injury. Furthermore, the penumbra tissue was included at the edge of lesion in 1-week and 2-week post-injury groups. In the 2-week injury group, the lesion was a cystic cavity containing cell debris and macrophages. Cell loss in the lesion increased over time until a cavity formed by 6-week post-injury. Furthermore, the irregular shape of spinal cord transverse section was noticed in 6-week post-injury group. In control groups, there were no histological evidences to suggest any lesion in the H&E sections of sham or normal control spinal cords. Moreover, the H&E image from the 6-week sham animal was used to represent control spinal cords in Figure 1A.

The injured area as a percentage of total cross sectional area was measured at the epicenter of injury in each spinal cord (Figure 1C). The mean injury percentage at the epicenter of injury ranged from 31.74 ± 7.83 to 70.16 ± 17.24%. There were significant differences in the injury percentage at the epicenter of injury between groups (ANOVA, *p* < 0.01). The injury percentage at the epicenter of injury in 24 h post-injury group was significantly higher than those in 1-week, 2-week and 6-week post-injury groups (Bonferroni *post hoc* comparisons, *p* < 0.05). There were no significant differences detected in the percentage of the injured area at the epicenter between 1-week, 2-week and 6-week injury groups. The mean percentage of the injured area at the epicenter for 1-week, 2-week and 6-week post-injury groups was 34.32 ± 3.67%.

### Cellular Responses

#### Nestin

**Figure 2:**
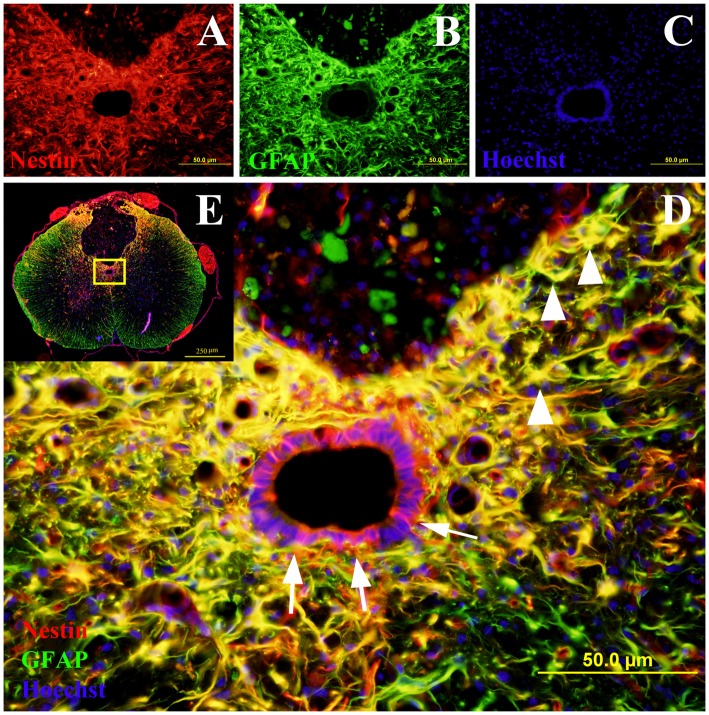
**Co-labeling of Nestin and glial fibrillary acidic protein (GFAP) in rat spinal cord transverse sections.** The Nestin positive cells were shown in red fluorescence **(A)**, while the GFAP was stained in green fluorescence **(B)**. All cell nuclei were counterstained in blue **(C)**. When the images were merged, the cells co-expressing Nestin and GFAP were shown in yellow at 400× magnification **(D)**. These study sites (the central canal and the lesion edge) were located in the yellow box area of the spinal cord transverse section at 40× magnification **(E)**. All the Nestin positive cells at the central canal were not co-labeled with GFAP (arrows). The cells at the lesion edge co-expressed Nestin and GFAP (arrowheads).

Nestin positive cells at the central canal consisted of intense cytoplasmic staining of ependymal cells with long basal processes (Figure 2). Nestin positive ependymal cells were seen in all the injury groups and only occasionally in the control groups. There was also increased Nestin staining at the lesion edge in the spinal cords of all the injured groups but not in comparative regions in non-injured controls. Occasional Nestin positive cells were seen in spinal cord blood vessels of all animals regardless of injury status (for example Figure 4A, 6-week sham).

#### Central Canal

The quantities of Nestin positive cells at the central canal were compared at different time points following mild contusion SCI in rat, along with sham and control groups (Figure 2). The central canal was identified by the histological morphology of ependymal cells in the round to oval arrangement. The central canal at the epicenter of injury was completely fragmented and not able to be identified due to the mild contusion injury. In injury groups, there were no significant differences in Nestin immunoreactivity at the central canal between rostral and caudal sections. In sham and control groups, the Nestin positivity at the central canal had no significant differences amongst the rostral, epicenter and caudal sections. Therefore, the mean GSV of Nestin immunoreactivity on rostral and caudal sections were used together in injury groups for further analysis, and those on rostral, epicenter and caudal sections were jointly considered in sham and control groups for statistic tests.

**Figure 3:**
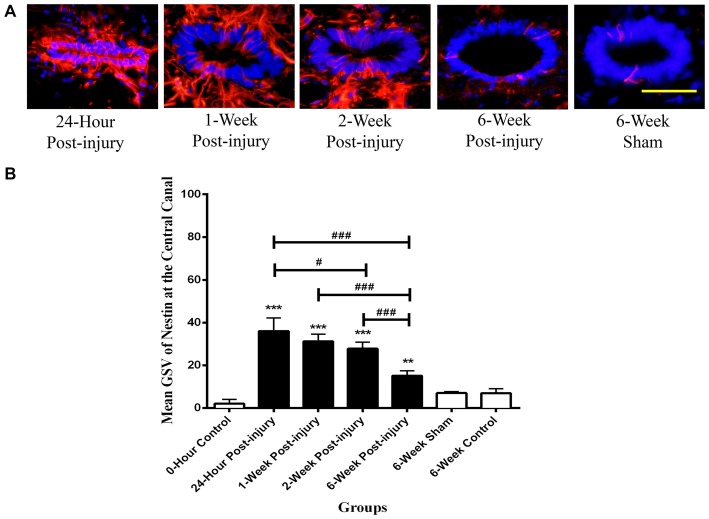
**Nestin positive cells at the central canal of rat spinal cord transverse sections. (A)** Nestin positive cells were shown in red fluorescence, and the cell nuclei were counterstained in blue. Scale bar: 25 μm. **(B)** Nestin immunoreactivity at the central canal of rat spinal cord transverse sections. The Nestin positivity peaked at 24 h post-injury, followed by a decreased trend in the Nestin immunoreactivity with the increasing post-injury time. Data are presented as mean ± SD. Statistically significant differences were shown compared to the 6-week sham group (**p < 0.01, ***p < 0.001). Statistically significant differences were revealed between groups (^#^*p* < 0.05; ^###^*p* < 0.001).

The Nestin positivity at the central canal peaked at 24 h post-injury and decreased with the increasing post-injury time in injury groups (Figure 3). There were significant differences detected in the Nestin immunoreactivity at the central canal between injury groups (ANOVA, *p* < 0.001) but not sham and control groups (Bonferroni *post hoc* comparisons, *p* > 0.05), so the 6-week control group was used as a standard for all subsequent statistical analysis. The Nestin immunoreactivity in all injury groups were significantly higher than that in 6-week control group (Bonferroni *post hoc* comparisons, *p* < 0.05). Moreover, the Nestin immunoreactivity at 24 h was two-fold higher than that at 6 weeks post-SCI. Counts of ependymal cell nuclei in the central canal epithelium in normal, sham and at different time points post-injury showed no significant differences. This indicates that there was no overall increase in ependymal cell numbers.

#### Lesion Edge

**Figure 4:**
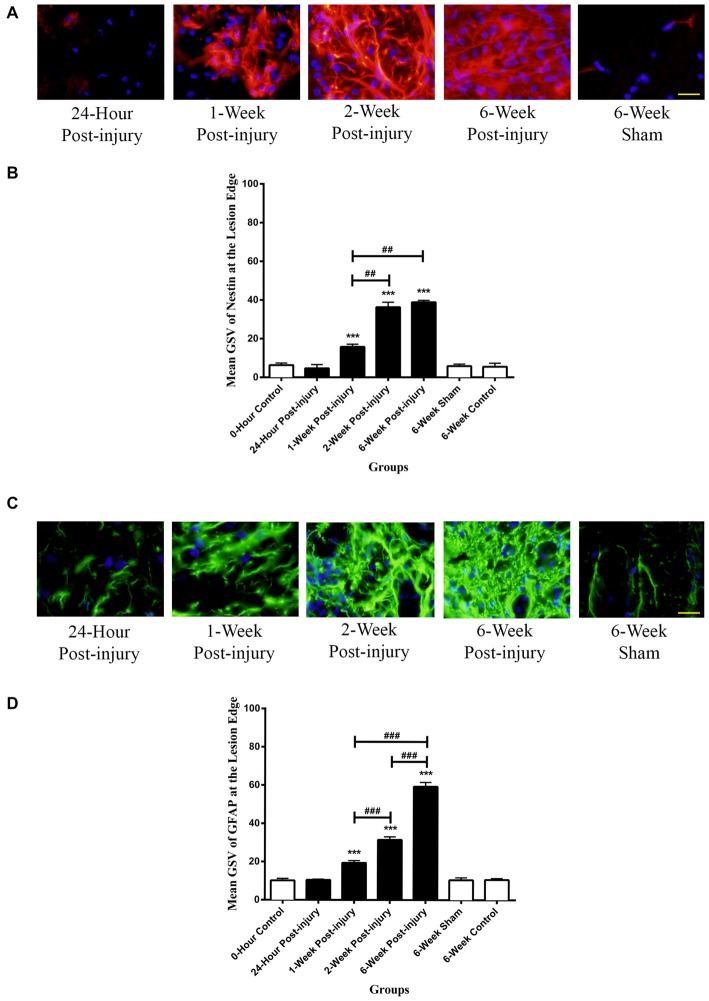
**Immunoreactivity of Nestin and GFAP at the lesion edge of rat spinal cord transverse sections. (A)** Nestin positive cells were shown in red fluorescence, and the cell nuclei were counterstained in blue. Scale bar: 25 μm.** (B)** The Nestin positivity significantly elevated at 1-week post-injury and kept increasing to a maximum at 6 weeks post-injury. **(C)** GFAP positive cells were shown in green fluorescence, and the cell nuclei were counterstained in blue. Scale bar: 25 μm. **(D)** The GFAP positivity significantly elevated at 1-week post-injury and kept ascending to the maximum at 6 weeks post-injury. All data are presented as mean ± SD. Statistically significant differences were shown compared to the 6-week sham group (***p < 0.001). Statistically significant differences were revealed between groups (^##^*p* < 0.01, ^###^*p* < 0.001).

The Nestin positivity at the lesion edge was compared between different time points following mild contusion SCI in rat, along with sham and control groups. In sham and control groups, blood vessels were stained as Nestin positive, and a population of cells at the lesion edge in injury groups showed distinct Nestin positivity (Figure 4A).

There were no significant differences between the GSV for Nestin intensity in the rostral, epicenter and caudal sections, thereby an overall GSV was used for statistical analysis. The Nestin positivity at the lesion edge was remarkably up-regulated from at 1 week post-injury and continuously increased to the maximum at 6-week post-injury (Figure 4B). The Nestin immunoreactivity at the lesion edge was significantly different between groups (ANOVA, *p* < 0.001). There were no significant differences in Nestin positivity between sham and control groups (Bonferroni *post hoc* comparisons, *p* > 0.05), so the 6-week control group was used as a standard for all subsequent statistical analysis. The Nestin positivity in 1-week, 2-week and 6-week post-injury groups was significantly higher than that in 6-week control group respectively (Bonferroni *post hoc* comparisons, *p* < 0.001). Furthermore, the 2-week and the 6-week post-injury groups had a significantly larger Nestin positivity compared to 1-week post-injury groups respectively (Bonferroni *post hoc* comparisons *p* < 0.01). The Nestin immunoreactivity at the lesion edge in 6-week post-injury group appeared to be greater than that in 2-week post-injury group, but not statistically significant (Bonferroni *post hoc* comparisons, *p* > 0.05).

#### GFAP

GFAP positive cells were observed in all the spinal cord sections and typically had the morphology of astrocytes with multiple long processes. White matter regions had increased GFAP staining compared to gray matter in all the control cords. No GFAP staining was seen in ependymal cells lining the central canal in any of the spinal cords.

#### Lesion Edge

There was intense GFAP staining of cells at the lesion edge for all injured spinal cords (Figure 4C). GFAP positive cells tended to be larger and had thicker processes than those seen in controls and in the surrounding tissue. It was difficult to identify individual cells due the overlap of cells and processes.

There were no significant differences in GFAP intensity in the rostral, epicenter and caudal sections so the average staining intensity for each spinal cord was used for all statistical analysis. The GFAP at the lesion edge was significantly increased from 1-week post-injury and continuously rose to the maximum at 6-week post-injury (Figure 4D). There were no significant differences in GFAP positivity between sham and control groups so the 6-week control group was used as a standard for subsequent analysis. The GFAP immunoreactivity in 1-week, 2-week and 6-week post-injury groups was significantly higher than that in 6-week control group respectively (Bonferroni *post hoc* comparisons, *p* < 0.001). Moreover, the GFAP positivity in 6-week post-injury group was three-fold higher than that in 1-week injury group and two-fold larger than that in 2-week injury group (Bonferroni *post hoc* comparisons, *p* < 0.001).

#### Nestin/GFAP Double Labeling

The co-labeling of Nestin and GFAP was performed to determine whether the Nestin positive cells co-expressed GFAP (Figure 2). The ependymal cells of the central canal did not co-express Nestin and GFAP. However, in the injury groups there were many cells at the edge of the lesion were double labeled with GFAP and Nestin (Figure 2).

**Figure 5:**
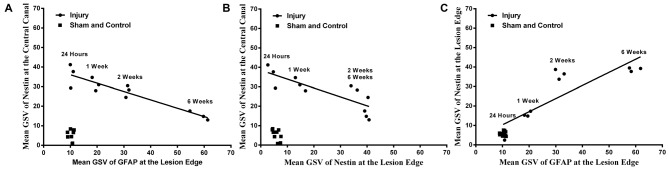
**Correlations between Nestin and GFAP immunoreactivity.** Nestin immunoreactivity at the central canal was found to decline with **(A)** GFAP immunoreactivity at the lesion edge (Pearson’s correlation, *r* = −0.871, *p* < 0.001) and **(B)** Nestin immunoreactivity at the lesion edge (Pearson’s correlation, *r* = −0.786, *p* < 0.001) respectively. **(C)** Nestin immunoreactivity was proportionally increased at the lesion edge with the GFAP immunoreactivity at the lesion edge (Pearson’s correlation, *r* = 0.900, *p* < 0.001).

It was found that there was a strong negative correlation between Nestin immunoreactivity at the central canal and GFAP immunoreactivity at the lesion edge (*r* = −0.926, *p* < 0.001), as well as the Nestin immunoreactivity at the lesion edge (*r* = −0.818, *p* < 0.01, Pearson’s correlations; Figure 5). Furthermore, there was a directly proportional relationship found between Nestin and GFAP immunoreactivity at the lesion edge (Pearson’s correlation, *r* = 0.867, *p* < 0.001).

## Discussion

The current study demonstrated the temporal response of endogenous NPCs following a mild SCI in rats. The contusion model described in this study led to the expected pattern of injury as reported by several groups (Basso et al., 1996; Mothe and Tator, 2005; Horky et al., 2006). At 24 h post-injury the tissue showed histological features consistent with an acute or primary SCI, for example, neutrophil infiltration, necrosis, hemeorrhage and axon swelling. By 6 weeks the tissue damage had developed into a cystic cavity (Li et al., 1999; Grossman et al., 2001; Westergren et al., 2001; Maier and Schwab, 2006).

The Nestin positive ependymal cells described in our study showed the well documented characteristics of NPCs reported by other groups (Frisén et al., 1995; Meletis et al., 2008; Cizkova et al., 2009; Barnabé-Heider et al., 2010). The up-regulated Nestin immunoreactivity in the ependymal layer of the central canal after injury has also been previously described (Yamamoto et al., 2001; Azari et al., 2005; Mothe and Tator, 2005; Cizkova et al., 2009). This population of cells at the central canal were Nestin positive and GFAP negative indicating NPCs rather than activated astrocytes (Lin et al., 1995; Johansson et al., 1999; Namiki and Tator, 1999). Although Nestin was not shown in control groups previously (Namiki and Tator, 1999; Mothe and Tator, 2005), we did see very occasional Nestin positive ependymal cells in the sham and control groups, suggesting that a limited number of quiescent endogenous NPCs do reside in the spinal cord. They probably function in maintaining homeostatic turnover of neural cells in normal adult spinal cords (Shibuya et al., 2002; Blasko et al., 2012). There were also a limited number of Nestin positive cells lining the blood vessels in the normal spinal tissue (Mokrý et al., 2004).

In our study Nestin staining was found to be highest in the ependymal cells lining the central canal at 24 h and then gradually declined over time. This is similar to the peak time reported previously by some authors (Namiki and Tator, 1999; Horky et al., 2006) but earlier than the peak at 3–7 days reported by others (Shibuya et al., 2002; Mothe and Tator, 2005; Cizkova et al., 2009; Hofstetter et al., 2005; Foret et al., 2010). It may be these authors were measuring specific populations of NPCs in different areas of the ependymal layer and sub-ependymal regions. In our study, we were unable to label proliferating cells specifically with either Bromodeoxyuridine (BrdU) or Ki67 and therefore could not determine whether increases in Nestin staining was due to cell proliferation and migration or increased Nestin expression within individual cells. We did not observe large numbers of Nestin positive cells in the paranchyma adjacent to the central canal at the early time points, nor were there any differences in total ependymal cell numbers between the controls and SCI cords at any time, suggesting an increase in Nestin expression rather than an increase in cell number, or large numbers of migrating cells. Other authors are confident that the peak Nestin expression in ependymal cells in fact represents localized proliferation followed by migration from the central canal into the sub-ependymal area. This migration has been shown by Mothe and Tator (2005) who intraventricularly inject 1,1′-dioctadecyl-6,6′-di(4-sulfophenyl)-3,3,3′,3′-tetramethylindocarbocyanine to label ependymal cells for monitoring their migration along with the immunohistochemistry staining of BrdU and Ki67 to detect their proliferation. This was also supported by Barnabé-Heider et al. (2010) who used BrdU to label the proliferating cells in transgenic mice for tracking the ependymal cells, and Horky et al. (2006) who utilized retroviral vectors to trace the migration of endogenous NPCs in conjunction with BrdU for examining their proliferation status. In the studies that showed cell proliferation (Yamamoto et al., 2001; Mothe and Tator, 2005; Cizkova et al., 2009) it can be seen that there were relatively few BrdU positive nuclei in ependymal cells that are actually also Nestin positive indicating that while cell proliferation occurs there is also a marked up-regulation of Nestin protein in the non-proliferating ependymal cells post-injury, which is similar to what we found.

Astrogliosis was indicated by the increase in intensity of GFAP staining and by hypertrophy of astrocyte processes at the lesion edge (Pekny et al., 2014). In the current study astrogliosis was shown to increase above baseline levels by 1 week post-injury and steadily increase for the 6-week post-injury period, as has been previously decsribed (Frisén et al., 1995; Horky et al., 2006). Recent studies have suggested that astrogliosis forms as early as 3–4 days post-injury (Tian et al., 2007; Tysseling-Mattiace et al., 2008). However, we could not confirm this, due to lack of post-injury time points between 1 day and 1 week.

Recently, reactive astrocytes, an important element of astrogliosis, were recognized by double labeling of GFAP and Nestin, which demonstrated the capability of reactive astrocytes to actively proliferate (Shibuya et al., 2002; Kozlova, 2003; Faulkner et al., 2004; Barnabé-Heider et al., 2010; Hu et al., 2010). As all the Nestin positive cells at the lesion edge co-expressed GFAP, Nestin immunoreactivity was utilized to investigate the response of reactive astrocytes following SCI in our study. The reponse of reactive astrocytes was not detectable at 24 h post-SCI, as shown in the early studies (Frisén et al., 1995; Shibuya et al., 2002). The maxium response of reactive astrocytes at the lesion edge was seen at 2 and 6 weeks post-injury as supported by Frisén et al. (1995). However, Shibuya et al. (2002) observed the strongest response of reactive astrocytes at 1 week post-injury, as a result of the examination of reactive astrocytes across the whole spinal cord transverse sections. Although from the traditional perspective, the glial scar largely composed of astrocytes was detrimental for neuroregeneration by preventing remyelination and regrowth of axons, the reactive astrocytes in glial scar were found to be beneficial for neural repair (Faulkner et al., 2004; Okada et al., 2006; Wang et al., 2011). As has been well documented, the reactive astrocytes in the glial scar lining the lesion edge separated the damaged tissue, resulting in the restriction of inflammation and oedema spreading to intact tissue (John et al., 2003; Faulkner et al., 2004; Okada et al., 2006). An earlier study also suggested that reactive astrocytes played a protective role by preventing the accumulation of extracellular glutamate, released from injured neurons, which could damage surrounding healthy neural cells (Bush et al., 1999). Conversely, Ikeda and Murase (2004) argued that the reactive astrocytes were adverse for neuroregeneration by generating and secreting nitric oxide, a neurotoxin that damaged local neural cells contributing to the secondary injury (Kozlova, 2003; Barnabé-Heider et al., 2010). The reactive astrocytes in astrogliosis were also reported to inhibit axonal regeneration and oligodendrocytes differentiation from precursor cells, leading to remyelination failure (Ikeda and Murase, 2004; Hu et al., 2010; Wang et al., 2011).

Several authors have suggested that NPCs at the central canal upregulate Nestin expression and migrate towards the lesion edge where they differentiate into activated astrocytes and contribute to the glial scar (Mothe and Tator, 2005; Horky et al., 2006; Ke et al., 2006). Furthermore, Barnabé-Heider et al. (2010) analyzed the origin of newly formed astrocytes at 2 weeks and 4 months post-SCI via transgenic mice and concluded that new astrocytes in the injured area came from both endogenous NPCs and astrocyte self-renewal. Foret et al. (2010) observed the increased GFAP expression in endogenous NPCs in the sub-ependymal area of the central canal 1 week after SCI, suggesting that the endogenous NPCs derived from the central canal mainly differentiated into astrocytes. While we saw the correlations that both Nestin and GFAP were increased at the lesion edge over the same time period, and there were many co-labeled cells, we did not see any evidence in the current study to suggest a direct result of migration and differentiation of NPCs from the central canal.

The current study agrees with the majority of the literature that NPCs located at the central canal are upregulated very early after SCI, certainly within 1 week, and most likely within 24 h, as shown by the Nestin immunoreactivity. The astrocyte response at the injury site, as indicated by increased GFAP and Nestin staining occurs from 1 week post-injury and continues for at least 6 weeks. Many studies concur that a reduction in astrogliosis with a simultaneous survival of neurons will be beneficial to spinal cord repair and recovery. The sequence of cellular responses and the time frame shown in this study indicate that if a reduction in astrogliosis is to be targeted as part of a treatment strategy there is a relatively short window of opportunity to attempt cellular manipulation. Whether NPCs migrate from the central canal and differentiate into astrocytes, or whether the astrocytic reaction is a localized response at the lesion site it remains true that there is only a short time frame in which to alter or reduce astrogliosis. The question of how this might be managed in the context of an acute SCI remains to be answered and there is still a need for a viable strategy to circumvent the inflammatory cascade that occurs during at the same time period.

## Author Contributions

YM participated in the animal surgeries, completed all of the immunohistology, imaging and analysis. He undertook most of the manuscript preparation. KM participated in the animal surgeries and preparation of tissue for histology and immunohistochemistry, and assisted in manuscript preparation. CAG undertook the animal surgeries and supervised all other aspects of the study including tissue analysis and manuscript preparation.

## Conflict of Interest Statement

The authors declare that the research was conducted in the absence of any commercial or financial relationships that could be construed as a potential conflict of interest.
